# Polyunsaturated Fatty Acids and Modulation of Cholesterol Homeostasis in THP-1 Macrophage-Derived Foam Cells

**DOI:** 10.3390/ijms11114660

**Published:** 2010-11-17

**Authors:** Masoud Salehipour, Ebrahim Javadi, Javad Zavvar Reza, Mahmoud Doosti, Shahla Rezaei, Malihe Paknejad, Naser Nejadi, Mansour Heidari

**Affiliations:** 1 Department of Medical Biochemistry, Faculty of Medicine, Tehran University of Medical Sciences, Tehran, Iran; E-Mails: salehipour_masoud@yahoo.com (M.S.); Doostimd@sina.tums.ac.ir (M.D.); shahla.rezaei@gmail.com (S.R.); paknejadma@tums.ac.ir (M.P.); naser@gmail.com (N.N.); 2 Department of Medical Biochemistry, Faculty of Medicine, Shahid sadoughi University of Medical Sciences, Yazd, Iran; E-Mail: jzavar@razi.tums.ac.ir; 3 Department of Medical Genetics, Faculty of Medicine, Tehran University of Medical Scienses, Tehran, Iran; E-Mail: Mheidari@sina.tums.ac.ir

**Keywords:** THP-1, conjugated linoleic acid, α-linlenic acid, eicosapentaenoic acid, liver X receptor α, ATP-binding cassette A1

## Abstract

Transformation of macrophages to foam cells is determined by the rates of cholesterol uptake and efflux. This study uses a real time RT-PCR technique to investigate the role of conjugated linoleic acid (CLA), α-linolenic acid (ALA) and eicosapentaenoic acid (EPA) in the regulation of the ATP-binding cassette A1 (ABCA1) and liver X receptor α (LXR) genes, which are involved in cholesterol homeostasis. Accordingly, these fatty acids significantly reduced the total, free and esterified cholesterols within the foam cells. While the expression of the ABCA1 and LXRα genes was increased in the presence of the pharmacological LXRα ligand, T0901317, their mRNA expression was not significantly affected by CLA, ALA and EPA. These results suggest that although polyunsaturated fatty acids have an effect on cholesterol homeostasis, they cannot change the expression of the ABCA1 and LXRα genes. Alternatively, several other genes and proteins may be involved.

## 1. Introduction

Accumulation of cholesterol in the walls of arteries provides the possibility of the uptake of modified low-density lipoprotein (m-LDL) by macrophages and hence, promotes a local pro-inflammatory response leading to a pathologic condition known as atherosclerosis [1]. The balance of cholesterol uptake and efflux in macrophages is a determining factor towards the formation of foam cells, which play a key role in the initiation and progression of atherosclerosis. Numerous genes and proteins, such as ATP-binding cassette A1 (ABCA1), liver X receptors (LXRs), sreol regulatory element binding proteins (SREBPs) and Peroxisome proliferator-activated receptors (PPARs), are involved in the process of foam cell formation, and study of these factors and their regulatory mechanisms is of great value for the prevention of cardiovascular disease (CVD). ABCA1 belongs to the large ATP-binding cassette transporter family. These transmembrane proteins transport cholesterol and phospholipids to lipid-free or lipid-poor apolipoprotein A-I (apoA-I). In fact, ABCA1, whose function is defective in Tangier disease, is a pivotal protein in regulation of plasma HDL-cholesterol levels and cellular cholesterol homeostasis [2–5].

Clinically, patients who are homozygous for Tangier disease accumulate macrophage-derived foam cells in various tissues. Several independent studies have demonstrated that ABCA1 is directly regulated by LXRs [6–9]. Also, several factors such as n-3 polyunsaturated fatty acids (PUFAs) have been shown to be able to regulate the expression of the ABCA1 gene, and hence, affect the cellular cholesterol efflux [10]. ω-3 PUFAs are also known to confer various health benefits, including increased insulin signaling [11], enhanced immune response [12,13], decreased plasma lipid levels [14,15], and finally decreased incidence of lung [16] and cardiovascular [17–19] diseases. In addition, conjugated linoleic acids (CLAs) are new potent anti-atherogenic dietary fatty acids in animal models of atherosclerosis and are capable of activating PPARs *in vitro* and *in vivo* [20]. Hu *et al.* showed that ω-3 PUFA can inhibit cholesterol efflux in THP-1 macrophage foam cells by promoting degradation of ABCA1 protein [21].

Liver X receptors (LXRα and LXRβ) are class II nuclear receptors that directly bind to the nucleotide repeats (LXRE sequence) as a heterodimer with Rethinoid X Receptor (RXR) [22]. Oxysterols, like 22(R)-hydroxycholesterol and 24,25-epoxycholesterol, have been shown to bind and activate the LXRs [23–25]. This activation leads to the binding of LXR/RXR heterodimers to LXRE sequences within the promoters of genes whose products are involved in hepatic bile acid synthesis, e.g., 7-hydroxylase (Cyp7A), and hence the main route for elimination of cholesterol from the body is activated. Moreover, LXRα limits the accumulation of cholesterol esters by inducing the expression of the ABCA1 and ABCG1 genes [6,9,26,27]. Joseph *et al.* showed that activation of LXR inhibited the development of atherosclerosis in mice [28] and some studies proposed the stimulant role of unsaturated fatty acids (USFAs) for LXRα but not LXRβ in cultivated hepatoma cells [29]. However, in contrast to these reports, other researchers believe that USFAs suppress the expression of the LXR gene [30]. Furthermore, PUFAs are found to compete with oxysterols for binding to LXRα, and thereby to suppress oxysterol-induced upregulation of SREBP-1c [24]. In the whole cell, it has been shown that eicosapentaenoic (EPA) and docosahexaenoic (DHA) acids decrease the cellular content of cholesterol-ester (CE) to approximately 50%, while this rate of decrease for some other fatty acids, like ω-3 linolenic, ω-6 linoleic and arachidonic acid,s drops to around 16%, compared to palmitic, stearic, or oleic acids. In fact, EPA is a poor substrate for esterification that reduces the incorporation of oleic acid into CE by up to 50%, whereas arachidonic, palmitic, stearic, and linolenic acids don’t have such an effect [31]. Accordingly, when human fibroblasts were grown in the presence of EPA for five days, both FC and CE levels were reduced in comparison with control and linoleic acid-treated cells [32].

Collectively, based on the mentioned reports, the role of ABCA1 and LXR towards the formation of foam cells is still a matter of debate. Therefore, with the final aim of studying the effect of expression of these two genes on the formation of foam cells, this study sought to address the influence of several distinct polyunsaturated fatty acids upon the transcription level of ABCA1 and LXRα genes in macrophages and additionally upon the cholesterol homeostasis in foam cells.

## 2. Results and Discussion

### 2.1. Toxicology Assay

**Figure 1.**
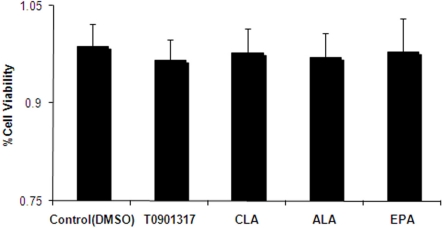
Assessment of cell viability by XTT assay. The presence of 100 μM conjugated linoleic acid (CLA), α-linolenic acid (ALA) and eicosapentaenoic acid (EPA) and 1 μM T0901317 was not toxic to the THP-1 cells, in comparison with the control group (DMSO). Data represent mean ± SD of at least three experiments performed in triplicate.

As the first step, we sought to confirm that the selected fatty acids have no toxicity on the THP-1 cells. To this end, XTT assay was performed in the presence of the different fatty acids CLA, ALA and EPA, in addition to T0901317 as the LXRα ligand. The results showed that fatty acids and T091317 have no adverse effect on the viability of THP-1 cell line, in comparison with control group (Figure 1).

### 2.2. Effect of Fatty Acids Treatment on the Expression of LXRα and ABCA1 Genes in THP-1-Derived Macrophage Foam Cells

**Figure 2.**
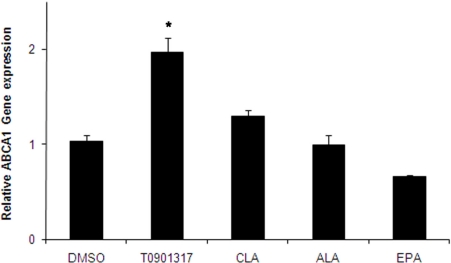
Effects of fatty acids (100 μM) and T0901317 (pharmacological LXRα ligand) on mRNA levels of the ABCA1 gene in THP-1-derived macrophage foam cells. THP-1-derived differentiated macrophages were initially pretreated for 24 h with either fatty acids or T0901317. Cells were then loaded with 50 μg/mL Ac-LDL in SFM medium for 48 h to induce foam cell transformation and incubated in the presence of fatty acids or T0901317 for a further 48 h. mRNA levels were analyzed by cyber green system. The values were normalized with respect to the level of β-actin mRNA. Results represent means ± SD from triplicate determinations, representative of 3 independent experiments compared with control. Statistical difference in comparison with the control group was analyzed by one-way ANOVA followed by Tukey multicomparison test. *P < 0.05.

At the next step, to provide the possibility to do our analysis on human macrophages, THP-1 cells, a human monocytic line were fully differentiated into macrophages by exposure to 100 ng/mL of phorbol 12-myristate 13-acetate (PMA) for 72 h. Before cholesterol loading, THP-1 macrophages were initially pretreated for 24 h with either ALA, c9,t11-CLA, or EPA (100 μM each) or T901317 (1 μM) as the control vehicle. Cells were then loaded with 50 μg/mL Ac-LDL in SFM medium for 48 h to induce foam cell transformation and incubated in the presence of fatty acids or pharmacological ligand of LXRα for a further 48 h. Results indicated that ABCA1 mRNA expression in THP-1-derived macrophage foam cells was not considerably affected by CLA, ALA and EPA fatty acid treatments (*P* = 0.82, *P* = 1.0 and *P* = 0.736, respectively), while the T901317 significantly increased the expression (Figure 2).

**Figure 3.**
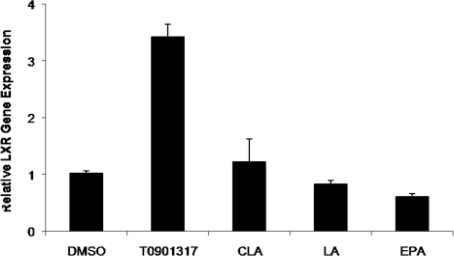
Effect of fatty acids (100 μM) and T0901317 (pharmacological LXRα ligand) on LXRα mRNA levels in THP-1-derived macrophage foam cells. THP-1-derived differentiated macrophages were initially treated for 48 h with fatty acids or T0901317. Cells were then loaded with 50 μg/mL of Ac-LDL in SFM medium for 48 h to induce foam cell transformation, and incubated in the presence of fatty acids or pharmacological ligand for a further 48 h. mRNA levels were analyzed by cyber green system. The values were normalized with regard to the β-actin mRNA level. Results represent means ± SD from triplicate determinations, representative of 3 independent experiments compared with control. Statistical difference in comparison with the control group was analyzed by one-way ANOVA followed by Tukey multicomparison test. *P < 0.05.

Furthermore, the mRNA expression level of the LXRα gene in THP-1-derived macrophage foam cells was not significantly changed by treatment with 100 μM CLA, ALA and EPA fatty acid compared to the cells treated with control DMSO, while T901317 significantly increased the expression (*P* = 0.65, *P* = 0.85 and *P* = 0.202, respectively) (Figure 3).

T0901317, the pharmacological ligand of LXR, was used as the positive control in these experiments. As expected, this compound significantly increased the mRNA expression levels of both the LXRα and ABCA1 genes in THP-1-derived macrophage foam cells, when compared with the control cells.

### 2.3. Distribution of Total Cellular Cholesterol in Cholesterol-Loaded Macrophages

**Figure 4.**
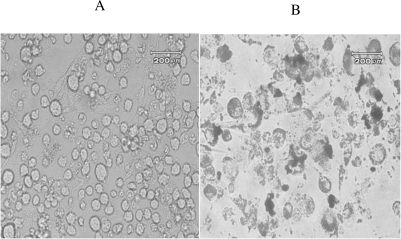
Transformation of macrophages to foam cells. THP1-derived macrophages (A) were incubated for 48 h in the presence of Ac-LDL-containing SFM medium (50 μg/mL). Lipid accumulation in THP-1-derived macrophages transformed them to foam cells that were identified by Oil Red O staining (B).

**Figure 5.**
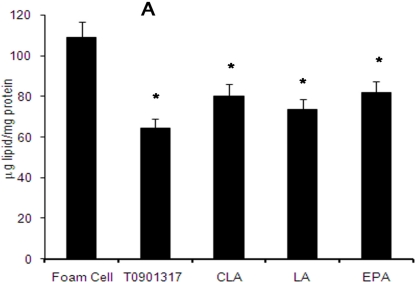

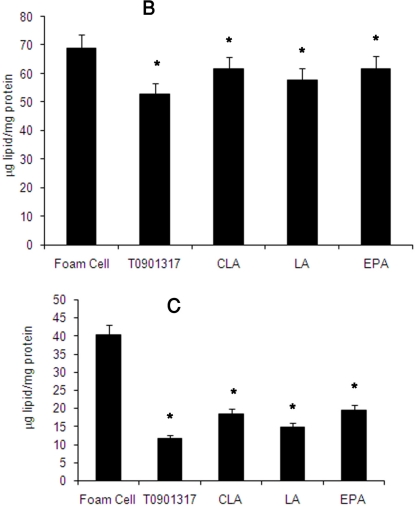
The effect of fatty acids and T901317 compound on the concentration of intracellular cholesterol as total cholesterol (A), free cholesterol (B) and esterified cholesterol (C) levels. THP-1-derived macrophages were cholesterol-loaded with Ac-LDL (50 μg/mL) for 48 h. Fatty acids and T0901317 were separately added to these cells 24 h before cholesterol-loading and thereafter every 24 h. TC and FC were enzymatically determined, and CE level was calculated as the difference between TC and FC amounts. Results show the mean ± SD of three independent experiments. Statistically significant differences between different groups are obtained by one-way ANOVA analysis followed by Tukey multicomparison test. Compared with untreated control group (foam cells), all treated groups illustrate a significant decrease (*p ≤ 0.05).

THP-1-derived differentiated macrophages, initially pre-treated with either fatty acids or T901317, were loaded with 50 μg/mL of Ac-LDL for 48 h and transformed to foam cells. Their increased cholesteryl ester content was reflected in the increased number of Oil Red-O staining lipid droplets (Figure 4). These THP-1-derived macrophage foam cells were then incubated with either 100 μM of fatty acids or 1 μM of T901317 for a further 48 h to determine the possible effects of fatty acids and T901317 on the concentration of intracellular total cholesterol (TC) and distribution of cholesterol forms as free cholesterol (FC) and cholesteryl ester (CE). The result demonstrated a significant decrease in intracellular concentration of TC, FC, and CE in comparison with not treated control foam cells (*P* ≤ 0.05) (Figure 5).

These results clarify the role of polyunsaturated fatty acids and activation of LXRα in the significant decrease of total cellular cholesterol in foam cells. Moreover, this study explained the effects of EPA, ALA and CLA on the cellular lipid distribution and the expression of ABCA1 and LXRα genes.

In the arterial intima, after monocytes are recruited, they obtain the morphological characteristics of macrophages and then accumulate cholesteryl esters (CE) as cytoplasmic droplets to guide foam cell formation. One of the most important factors in prevention of above phenomena is cholesterol efflux via ABCA1. Several independent studies have demonstrated that ABCA1 is directly regulated by LXRα [6–9] and that PUFAs are the activating ligands for LXRα. In the present study, we showed that PUFA has no significant effect on ABCA1 and LXR gene expression in human THP-1 macrophage-derived foam cells, however, incubation of THP-1 derived foam cells with fatty acids led to the reduction of TC, CE and FC levels. Of note, EPA and ALA non-significantly lowered the expression of the ABCA1 gene, while CLA showed a trend for higher expression (Figure 2). Similar results were observed in case of the mRNA expression of the LXRα gene as well (Figure 3). There are several conflicting data regarding the effect of PUFA on the transcription level of LXR: Uehara *et al.* reported no effect on the mRNA levels of LXRα or RXR [33], while other researchers demonstrated either the stimulatory [29] or suppressive [30] effects on the LXRα gene expression. Moreover, fatty acids have been shown to activate the PPARα and γ [34], which indirectly increases the ABCA1 gene expression by positive regulation of the LXRα gene.

The intracellular mechanisms of cholesterol homeostasis are complex and various factors are involved. As so, recent studies indicate that PPARα stimulates post-lysosomal mobilization of cholesterol [35] and unsaturated fatty acids activate the ABCA1 at the protein level and induce the LXR and ABCA1 genes to enhance their transcription levels [36]. Fatty acids are shown to activate PPARα and PPARγ, as well [37]. Accordingly, Hirakata *et al.* have proposed that PPARγ ligands reduce the CE accumulation in macrophages by induction of CEH (cholesterol ester hydrolase, whose product enzymatically hydrolyzes the CE to free cholesterol and fatty acids) and inhibition of ACAT-1 (acyl-CoA:cholesterol acyltransferase-1, whose product catalyzes cholesterol ester formation from cholesterol and fatty acyl-CoA) genes expression [38]. From the other side, Davis has shown that ω–3 polyunsaturated fatty acids significantly reduce the rate of cholesterol esterification in macrophages, most likely due to the inhibition of ACAT-1 [39]. Another factor involved is CD36, which is a multi-ligand scavenger receptor on the surface of monocytes/macrophages. This molecule causes the endocytosis of the modified LDL (m-LDL), which leads to the formation of foam cells. Interestingly, ω-3 fatty acids have been shown to reduce CD-36 expression at the gene and protein levels, which in turn prevents the formation of macrophage foam cells [40].

It is noteworthy that SR-B1, which belongs to the class B scavenger receptor family, has the capability to bind m-LDL, as well. This receptor has gained importance due to its role in reverse cholesterol transport, and since it is involved in cholesterol efflux in macrophages, it may be considered as a possible therapeutic intervention point for researchers. Studies demonstrate that SR-B1 is a target for being regulated by PPARs [41]. As mentioned, PUFAs can active PPARs gene expression and therefore, they may indirectly increase the expression of SR-B1 and enhance the cellular cholesterol efflux in macrophages-derived foam cells.

Taken together, our results - in accordance with the mentioned findings - suggest that free PUFAs possibly reduce the CE concentration within the foam cells and hence, can affect the development of arthrosclerosis. Moreover, we found that cholesterol homeostasis is not just affected by factors like ABCA1 and LXR, but the complicated interaction of different factors, which were mentioned above, as well. Therefore, investigating the effect of fatty acids on one gene does not result in a distinct conclusion, as the available reports also rely on the interaction of multiple factors, which should be studied in more detail.

## 3. Materials and Methods

### 3.1. Chemicals

T0901317 (pharmacological LXRα ligand), phorbol 12-myristate 13-acetate (PMA), dimethyl sulfoxide (DMSO), linoleic acid, eicosapentaenoic acid and conjugated linolenic acid were obtained from Sigma-Aldrich, USA. Phosphate buffered saline (PBS) tablets were obtained from Takara, Japan. Reagents for RNA extraction and reverse transcription were obtained from Qiagen, Germany. All other chemicals were obtained from Sigma-Aldrich, USA.

### 3.2. Cell Culture

Human monocytic THP-1 cell line was obtained from national cell bank of Iran (Pasteur institute, Iran), cultured at 1.0 × 10^6^ cells per well in 6-well plates and maintained in RPMI 1640 medium (Invitrogen, USA) supplemented with 10% heat inactivated fetal bovine serum (FBS), sodium pyruvate, 1 mM l-glutamine, 30 μL/mL amphotricin B, 100 μg/mL streptomycin, 100 U/mL penicillin and 100 μM 2-mercaptoethanol at 37 °C in a humidified atmosphere of 5% CO_2_. To differentiate monocytes into macrophages, THP-1 cells were washed with serum free RPMI 1640, resuspended in SFM (serum free medium) containing PMA (100 ng/mL) and cultured for 3 days [42].

### 3.3. Isolation and Acetylating of LDL

LDL was isolated from human plasma by density gradient ultracentrifugation at 200,000 g for 10 h at 4 °C, as previously described [43]. LDL-protein concentration was determined using Bradford assay. Isolated LDL was additionally acetylated by saturated sodium acetate and acetate anhydride [44].

### 3.4. Oil Red-*O* Staining

For transformation of macrophages to foam cells, THP1-derived macrophages were incubated for 48 h in the presence of Ac-LDL-containing SFM medium (50 μg/mL). Cells were fixed and stained with Oil Red O, as previously described [45].

### 3.5. Fatty Acids and T0901317 Treatment

All fatty acids including Conjugated Linoleic Acid (CLA), α-Linolenic Acid (ALA), and Eicosapentaenoic Acid (EPA) in addition to T0901317 compound were prepared in DMSO. THP-1-derived macrophages were pretreated with fatty acids and T0901317 for 24 h (final concentration ≤0.01%) and then transformed to foam cells by incubation with 50 μg/mL of Ac-LDL.

### 3.6. RNA Isolation and Real-Time RT-PCR

Total cellular RNA was extracted using the RNeasy kit (Qiagen, Germany), according to the manufacturer’s protocol. cDNAs were synthesized from 1 μg of extracted RNA using quantitect reverse transcriptase kit (Qiagen, Germany). Constructed cDNAs were quantitatively PCR-amplified using SYBRgreen detection system in an ABI PRISM 7900HT instrument. The result for each template was normalized relative to the obtained amount for α-Actin expression.

### 3.7. Toxicology Assay

Viability of the cells treated with 100 μM fatty acid and 1 μM T0901317 was quantified spectrophotometrically by 2,3-bis(2-methoxy-4-nitro-5-sulfophenyl)-*S*-(phenylamino) carbonyl-2-tetrazolium hydroxide (XTT) assay (Sigma-Aldrich), as described by Scudiere *et al.* [46]. THP-1 macrophages were cultured with or without the fatty acids and T0901317 for 24 h in a 96-well microplate. After an appropriate incubation (37 °C, 4 h) with XTT, plates were shaken and absorbance was measured at 450 nm. Results were expressed as the percentage of cell viability with respect to the control negative absorbance (100% cell viability).

### 3.8. Intracellular Cholesterol Measurement

Differentiated THP-1 macrophages were cultured in SFM and pre-treated with fatty acids and T0901317 for 24 h. These cells were then treated with 50 μg/mL of ac-LDL and incubated in the presence of fatty acids or T0901317 for 48 h. After washing twice with PBS and homogenization in 200 μL of hexane-isopropanol (3:2) [47], the organic phase was used for free and total cholesterol measurements by enzymatic assays (Calbiochem, USA). Esterified cholesterol was also measured as the difference between total and free cholesterol. Cells were lysed with RIPA buffer (0.1% SDS, 100 mg/mL PMSF, 0.5% sodium deoxycholate, 10 mM sodium phosphate, 150 mM NaCl, 30 mg/mL aprotinin, 1 mM sodium orthovanadate and 50 mg/mL Dithiotrithol, pH 7.4) [48] and cellular protein was measured by Bradford assay [49]. Results were presented as μg lipids/mg cellular proteins.

### 3.9. Statistical Analysis

All results are presented as means ± S.D. Differences among groups were determined by one-way ANOVA, with Post-Hoc comparison by Tukey multiple comparison test. The level of significance for all statistical analyses was set at *p* < 0.05. Analyses were performed using SPSS on Windows software.

## 4. Conclusion

In conclusion, while this study suggests a role of fatty acids toward the reduction of cholesterol accumulation within the foam cells, it excluded the distinct role of ABCA1 and LXRα in this process. Therefore, it could be concluded that fatty acids might imply their effect through other pathways such as PPARs. The process of lipid metabolism within the foam cells seems to be very complicated, and this fact can explain the controversial results that are reported by different research groups. Accordingly, detailed evaluation of other involved genes and factors at the same time will be necessary.
